# Integrins in disguise - mechanosensors in *Saccharomyces cerevisiae* as functional integrin analogues

**DOI:** 10.15698/mic2019.08.686

**Published:** 2019-07-15

**Authors:** Tarek Elhasi, Anders Blomberg

**Affiliations:** 1 Dept. of Chemistry and Molecular Biology, Univ. of Gothenburg, Sweden.

**Keywords:** integrins, yeast, mechanosensors, stress-responses, starvation

## Abstract

The ability to sense external mechanical stimuli is vital for all organisms. Integrins are transmembrane receptors that mediate bidirectional signalling between the extracellular matrix (ECM) and the cytoskeleton in animals. Thus, integrins can sense changes in ECM mechanics and can translate these into internal biochemical responses through different signalling pathways. In the model yeast species *Saccharomyces cerevisiae* there are no proteins with sequence similarity to mammalian integrins. However, we here emphasise that the WSC-type (Wsc1, Wsc2, and Wsc3) and the MID-type (Mid2 and Mtl1) mechanosensors in yeast act as partial functional integrin analogues. Various environmental cues recognised by these mechanosensors are transmitted by a conserved signal transduction cascade commonly referred to as the PKC1-SLT1 cell wall integrity (CWI) pathway. We exemplify the WSC- and MID-type mechanosensors functional analogy to integrins with a number of studies where they resemble the integrins in terms of both mechanistic and molecular features as well as in the overall phenotypic consequences of their activity. In addition, many important components in integrin-dependent signalling in humans are conserved in yeast; for example, Sla1 and Sla2 are homologous to different parts of human talin, and we propose that they together might be functionally similar to talin. We also propose that the yeast cell wall is a prominent cellular feature involved in sensing a number of external factors and subsequently activating different signalling pathways. In a hypothetical model, we propose that nutrient limitations modulate cell wall elasticity, which is sensed by the mechanosensors and results in filamentous growth. We believe that mechanosensing is a somewhat neglected aspect of yeast biology, and we argue that the physiological and molecular consequences of signal transduction initiated at the cell wall deserve more attention.

## INTRODUCTION

The yeast *Saccharomyces cerevisiae* is an excellent test bed for the study of human proteins, and in a recent systematic, large-scale study it was found that roughly 50% of human proteins with homology to yeast proteins could compensate for the corresponding gene deletion in yeast [1]. This systematic study of human proteins in yeast revealed a high degree of conserved functions; in particular for genes involved in specific functional classes like central metabolism and lipid and sterol biosynthesis. Human proteins with no clear homology to yeast proteins can also be studied in this model system, and several important disease models of human genes have been developed and utilised for functional studies in yeast (the following reviews provide excellent overviews and a more complete list of the use of yeast in surrogate genetics related to human disease [2–4]). Thus, *S. cerevisiae* has proven its worth as a powerful tool in the discovery and study of mechanisms involved in a wide array of human diseases.

The strategy in yeast surrogate genetics is to learn about human proteins by studying their functions in yeast. However, in this review we will explore the opposite logic – to start with knowledge about a human protein and then seek out functionally equivalent analogues in yeast. We focus on integrins and integrin-signalling in humans and review the importance and functions of mechanosensor analogues in yeast. Integrin-mediated adhesion and signalling are probably the most important cell adhesion mechanisms for metazoan development. However, many of the components of the integrin adhesome evolved well before the origin of metazoans and fungi [5], and it has been suggested that several key components of the integrin adhesome have been lost independently in fungi and choanoflagellates. In this context, it is interesting to note that genome sequences from unicellular metazoan-related lineages have pushed the time of origin of gene families earlier believed to be metazoan-specific back into the Proterozoic period, e.g. tyrosine kinases [6], transcription factors [7], and cadherins [8].

We will start with a short overview of the function of integrins in humans and the evolution of integrin and integrin-like systems. We will then make connections to the mechanosensors in yeast and the various aspects of their molecular functions and biological roles, highlighting where we think they are similar to integrins. Finally, we will outline several hypotheses in relation to the cell wall in yeast and the role of the mechanosensors in transmitting cell wall changes to intracellular signalling. We think yeast mechanosensors deserve more attention and that future studies will most likely reveal novel biological activities, and it is possible that the knowledge generated in yeast will feed back to the human system to provide a better understanding of the complexity of mechanosensing and the function of integrins in humans.

## BACKGROUND TO INTEGRINS

### Integrins in mammals

The ability to sense mechanical stimuli is vital for all organisms. In mammals, mechanical stimuli are mainly sensed by transmembrane adhesion receptors called integrins that mediate cell adhesion to the extracellular matrix (ECM) or to neighbouring cells [9]. The extracellular domain of integrin interacts with extracellular protein ligands that bind specifically to integrin, while the intracellular domain acts as an attachment site for the internal cytoskeleton through actin-associated proteins, forming the focal adhesion complex (FAC) [10, 11]. In this way integrins physically link the ECM to the cytoskeleton in the interior of the cell [11]. In fact, the name “integrin” was given to these cell receptors because of their importance in the integration of the ECM and the cytoskeleton [12, 13]. Integrins are highly important proteins involved in a wide variety of processes such as the development of the immune system [14], apoptosis [15], and wound healing [16]. They are also functionally involved in many diseases such as cancer [17], diabetes [18], and Alzheimer's [19] as well as in aging [20].

**Figure 1:**
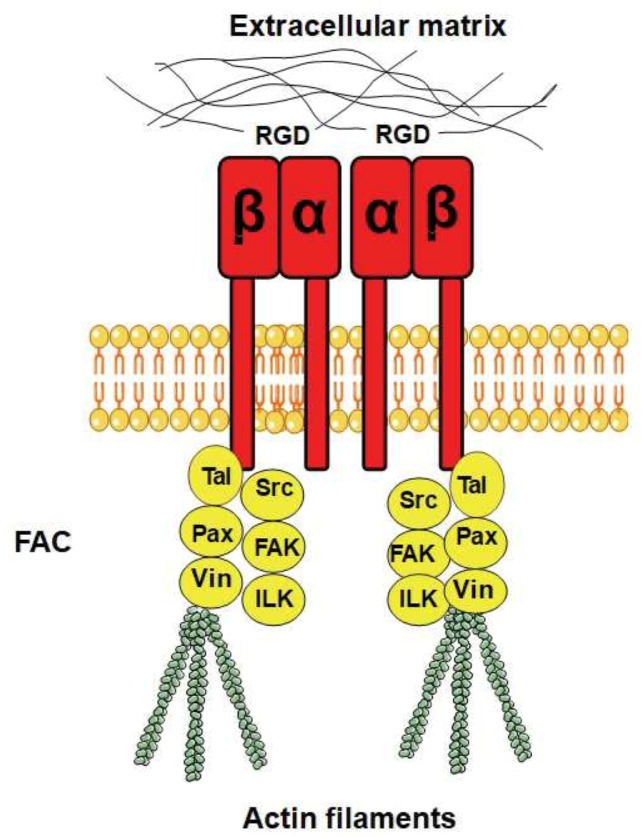
FIGURE 1: The overall structure of human integrin α and β and some of their downstream effectors. Binding of integrins (in red) to the ECM, e.g. to RGD motifs in ECM proteins, promotes integrin clustering and association with the actin cytoskeleton. Interactions with the down-stream effectors are mediated via integrin β. Intracellular effectors that are part of the focal adhesion complexes (FACs) include talin (Tal; an adapter protein), focal adhesion kinase (FAK; a non-receptor tyrosine kinase), paxilin (Pax; an adapter protein), Src-family kinase (Src; a tyrosine kinase), integrin-linked kinase (ILK), and vinculin (Vin).

Integrins are heterodimers composed of α and β subunits, which are non-covalently bound to each other [12]. In humans, there are 18 α subunits and eight β subunits that physically interact and form 24 different heterodimers [21]. Integrins are localised to the plasma membrane and are composed of a rather extensive extracellular domain that is about 20 nm long [22], one transmembrane domain with a single membrane-spanning helix, and a shorter intracellular domain **(Fig. 1)**. Integrin β subunits contain a number of rather well-studied domains, including the von Willebrand A (VWA) domain, an N-terminal PSI domain, and repeated EGF-like domains [23]. Many VWA domains bind metal ions via a sequence motif called the metal ion-dependent adhesion site (MIDAS) with the consensus sequence DXSX, and a number of human diseases arise from mutations in the VWA domain [24]. The majority of VWA domains are found in cell adhesion and ECM proteins [25], as well as in certain classes of intracellular proteins [24]. A plausible hypothesis for the function of the VWA domain is that it supports protein–protein interactions through the joint coordination of a metal ion with the MIDAS motif [26]. Although integrins are heterodimers, it has been suggested that β integrin is the principle subunit for the intracellular binding to the cytoskeleton and the extracellular binding to signalling molecules, while α integrin has a regulatory role in maintaining the proper conformation of β integrin for binding to its ligands [27].

### Integrin ligands

The different combinations of α and β subunits create different kinds of integrins with different ligand specificities, with some integrins binding to ECM proteins and others binding to cell surface receptors. Some integrins recognise the RGD motif on their ligands as an active site for binding **(Fig. 1)**, while other integrins recognise the LDV motif. The ECM ligands with RGD as the active integrin binding site include vitronectin, fibronectin, and fibrinogen, where vitronectin and fibronectin also contain an LDV domain. Integrins also bind to proteins with alternative binding motifs; for example, collagen has the GFOGER motif (where O is hydroxyproline) as its recognition site while the recognition site of laminin is not yet known [27]. Synthetic peptides that contain an RGD motif can inhibit integrin signalling by outcompeting RGD-containing ligands, and these peptides are widely used as integrin inhibitors and have been used to study integrin signalling in different organisms (see below).

### ECM and integrin signalling in mammals

In mammals, the nature of the ECM is of great importance, and integrins mediate the response to ECM stiffness that determines cell fate [28]. Cells remodel the ECM in response to the force applied to integrin-mediated adhesions. For example, when fibroblasts are stretched they activate the expression of genes coding for collagen, fibronectin, and metalloproteins and form a dense ECM that is rich in collagen [29]. This regulation occurs in many cases through signalling pathways downstream of integrins [30]. Integrin expression also changes according to ECM elasticity [31], and this elasticity probably causes conformational changes in integrins – and thus changes their ligand-binding properties – or exposes buried domains to their interacting proteins [32].

### Integrins and actin foci in mammals

FACs are specialised sites where clustered integrin receptors interact with the ECM on the outside of cells and with the actin cytoskeleton on the inside of cells **(Fig. 1)**. The FACs provide adhesion to the ECM and transmit mechanical tension generated within cells across the plasma membrane to the external environment. In addition, they act as scaffolds for many signalling pathways that are triggered by ligands binding to the integrins or by mechanical forces exerted on the cells [32, 33]. FACs are distributed in the cell in a punctate pattern [34], and the most important components of FACs are talin (an adapter protein), focal adhesion kinase (FAK, a non-receptor tyrosine kinase), paxilin (an adapter protein), Src-family kinase (another tyrosine kinase), integrin-linked kinase, and vinculin.

Talin was found to be instrumental for the activation of integrins and to enhance the association between integrins and the ECM [35]. It has been shown that the stretching of talin exposes multiple cryptic vinculin-binding sites that are normally (without the tension) buried within talin [36]. Given the prominence of talin and vinculin in FACs, this tension-induced interaction is likely a key factor for FAC assembly, growth, and function.

### Integrin signalling in mammals

When bound to an extracellular ligand, integrins bind intracellularly to many other proteins to form the FAC. When the FAC is formed, it activates diverse signalling components like the tyrosine kinase c-Jun N-terminal kinase, phosphatidylinositol 3-kinase (AKT), the GTPases of the RHO family [37], and the mitogen activated protein kinases (MAPKs) ERK and p38 [38].

The interactions between integrins and the ECM are important in order for the cell to adjust its intracellular activities in response to the surrounding microenvironment, and these interactions occur through two different mechanisms [39]. First, there are signals from the ECM to the cell (outside-in signalling). Here integrins sense a change in the ECM tension (if ECM is loose or stiff) and sense the dimensional changes in the ECM (increases in depth/volume). In addition to the response to the ECM dynamics, integrins also receive signals from antagonists like growth factor receptors, and they interact with other membrane proteins like tetraspanins that function as adaptors in recruiting other integrin interactors [40]. In the second type of interaction (inside-out signalling), cellular signals bind to the integrin cytopasmic domain and cause a conformational change that result in integrin activation [41].

## EVOLUTION OF INTEGRINS

The integrin α and β subunits exist throughout the metazoans. It is believed that integrins have an early origin, preceding the first metazoans, with some integrin domains identifiable even in bacteria [42, 43]. For example, proteins containing VWA domains are present throughout the eukaryotes (metazoa, fungi, plants, and protists) as well as in eubacteria and archaea. However, both integrin α and β, as well as several other components of the integrin adhesion complex, are absent from choanoflagellates and fungi and were presumably lost independently in these two lineages. However, integrin-like functions exerted by integrin functional analogues seem conserved even if integrin sequence homologues might be missing.

### Integrin-like proteins in plants

The cell wall is considered the ECM in plants [44], and similar to the animal ECM the plant cell wall contains mechanosensors that sense mechanical changes imposed on the cell wall from the surrounding environment. There are no integrin sequence homologues in plants, but experimental evidence – mainly based on immunoblotting techniques – suggests the existence of what are called "integrin-like proteins" and "integrin-like signalling" in plants. For example, monoclonal antibodies against chicken β1 integrin, human vitronectin, and human fibronectin recognised proteins on the surface of protoplasts isolated from onion epidermis and tobacco root cap [45, 46]. In addition, polyclonal antibodies against chicken β1 integrin detected proteins in the *Arabidopsis thaliana* plasma membrane [47].

Integrin inhibitor peptides containing RGD sequences have been exploited in plant studies to study integrin-like mechanisms. *Arabidopsis* protoplasts agglutinate when expressing ProNectin, a genetically engineered protein that contains 13 RGD sequences, and RGD peptides disrupt this adhesion [48]. In another study, application of RGD peptides to peas disrupted the cell wall-plasma membrane connection [49]. Overall, applying RGD-containing peptides to plant cells interferes with many physiological processes such as development [50], cytoplasmic streaming [51], gravisensing [52], root induction [53], and defence responses [54]. The RGD peptides seem to act on proteins at the plasma membrane-cell wall interface [54]. It has been proposed that these integrin-like proteins likely act as mechanosensors as part of the defence response, and that their attachment to receptors in the cell wall or changes in the mechanical property of the cell wall might warn the host plant about pathogen invasion [55]. In *A. thaliana*, the predicted structure of NDR1, a membrane protein involved in the defence response, showed that the core of NDR1 has strong 3-dimensional structural similarity with β integrin [56]. In addition, the *A. thaliana* genome codes for five proteins in the integrin-linked kinase family. Another important protein in FACs, FAK, has also been detected in plants [57]. Moreover, proteins with sequence similarity to filamin, α-actinin, integrin-linked kinase, and proteins with a LIM domain (which is important for protein association to the FAC) have been found in green algae [58]. In summary, it is likely that integrin-like proteins and integrin-like signalling exist throughout the plant kingdom.

### Integrin-like proteins in unicellular organisms

In immunoblotting experiments, integrin-like proteins have also been detected in oomycetes [59] and in the amoebae *Naegleria fowleri* and *Naegleria lovaniensis* [60]. Furthermore, the integrin-like protein β1*Eh*FNR was detected in *Entamoeba hystolytica* based on immunoblotting [61]. Despite the lack of sequence similarity between β1*Eh*FNR and mammalian integrin, β1*Eh*FNR shows functional similarity to integrins because it forms intracellular FACs. These complexes contain homologues to many mammalian FAC proteins such as actin, myosin I, myosin II, alpha actinin, and FAK [62] as well as paxilin and vinculin [63]. The *E. hystolytica* β1*Eh*FNR does not form a heterodimer like the mammalian integrins do, suggesting that integrin-like signalling can be mediated solely by the equivalent to the integrin β subunit.

### Integrin-like proteins in fungi and yeast

It was shown by applying RGD-containing peptides that integrin-like proteins in fungi are important for fungal germination during the pre-penetration stage [64]. Based on immunoblotting assays, integrin-like proteins were detected in the yeast form of *Histoplasma capsulatum* [65], and RGD peptides inhibited conidial adhesion and appressorium formation in *Magnaporthe oryzae* [66]. The conditional yeast pathogen *Candida albicans* binds to hyphae of human lymphocyte cells. This binding was shown to be inhibited by a variety of RGD-containing peptides; thus, indicating that some integrin-like proteins on *Candida* are involved in this yeast-human cell binding [67]. Furthermore, the adhesion of the *C. albicans* germ tube to purified human vitronectin was inhibited by monoclonal antibodies against integrins α5 and β3, thus showing that *Candida* contains some integrin-like proteins [68].

**Figure 2:**
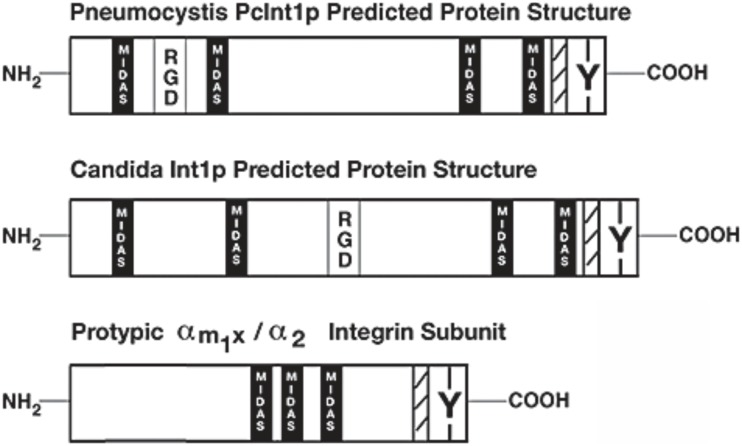
FIGURE 2: Integrin-like proteins in fungi share some of the overall structure and motifs with mammalian integrins. Schematic representation of integrin-like INT1 from *Candida albicans* and *Pneumocystis carinii*. Indicated are the approximate locations of some motifs – RGD (the integrin binding site), MIDAS (the metal ion-dependent adhesion site), Y (the conserved tyrosine-residue in the C-terminal part), and hatched marking (the transmembrane domain). A prototypical human integrin subunit is shown at the bottom. This figure is from [70] and is published with permission from John Wiley and Sons. Copyright © 2008, American Society for Microbiology.

The protein INT1/BUD4 in *C. albicans* has integrin-like motifs that are involved in integrin-ligand binding, and this protein localises to the cell surface [69]. It was also reported that the ascomycete fungus *Pneumocystis carinii* (a pneumonia-causing pathogen) expresses a protein with considerable structural similarity to INT1 [70] **(Fig. 2)**. The 1,006 amino acid *Pneumocystis* protein (pcINT1) has homology to the C-terminal region of *C. albicans* INT1 (31% sequence identity) and contains four MIDAS motifs (DXSX) required for adhesion and for coordination of divalent cations, a membrane spanning region, an internal RGD motif, and a specific tyrosine residue found in the cytoplasmic tails of some human integrin receptors and in INT1 from *C. albicans.* The internal RGD motif in INT1 might well just be a coincidence, because RGD is not found to be functional in human integrins. Furthermore, heterologous expression of pcINT1 in *S. cerevisiae* showed that pcINT1 was localised to the surface and that yeast cells subsequently gained the ability to bind to human fibronectin in a cation-dependent fashion. However, despite the presence of several integrin-like motifs none of the INT1 proteins are good sequence homologs to human integrins; the caINT1 and the human integrins have only roughly 20% overall sequence similarity (global alignment).

## MECHANOSENSORS IN *SACCHAROMYCES CEREVISIAE* – INTEGRINS IN DISGUISE

### No integrin homologues in *S. cerevisiae*

There are no sequence homologues to human integrins in the yeast *S. cerevisiae*. Blast searches with any of the 26 human integrin α or integrin β proteins do not yield any hits (E-value threshold 10^−5^) in the *S. cerevisiae* genome. However, the integrin-like INT1 proteins from *Candida* or *Pneumocystis* both have rather strong matches to the *S. cerevisiae* sequence homologue Bud4 (E-value of 10^−47^ and 10^−20^, respectively), but with homology only to a very distinct C-terminal region. Bud4 is an anillin-like protein that is involved in bud-site selection and the formation and disassembly of the double septin ring structure in *S. cerevisiae*. However, it has no membrane-spanning domain and consequently no extracellular region, and therefore does not have an overall integrin-like structure like INT1. Bud4 in *S. cerevisiae* is thus not believed to be functionally analogous to INT1 or to animal integrins.

### Mechanosensors in *S. cerevisiae* as functional integrin analogues

**Figure 3:**
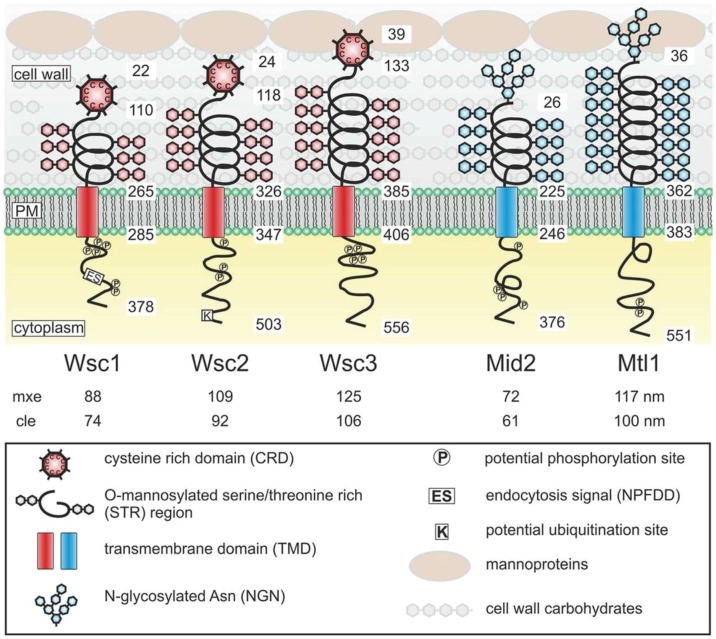
FIGURE 3: Structures and proposed functional domains of yeast mechanosensors. Protein domains are indicated in the legend box. PM, plasma membrane. Numbers indicate residues in the respective proteins counting from the N-termini. Numbers below the protein names indicate the estimated length of the extracellular sensor region. mxe, maximal possible extension. cle, computed live-cell extension. This figure is from [72] and is published with permission from the American Society for Microbiology. Copyright © 2015, American Society for Microbiology.

There is a group of proteins that act as mechanosensors in yeast and that activate different intracellular signalling pathways upon external perturbations of either the cell wall or the plasma membrane in much the same way as mammalian integrins. Various environmental cues recognised by these mechanosensors are transmitted by a conserved signal transduction cascade commonly referred to as the PKC1-SLT1 CWI pathway [71]. Similar to integrins, these mechanosensors in yeast consist of a long extracellular domain that is anchored to the ECM (i.e. the cell wall in the case of yeast), a single transmembrane domain that anchors these proteins in the plasma membrane, and a short C-terminal cytoplasmic tail that interacts with and activates intracellular signalling components **(Fig. 3)** [72]. Another similarity is that both human integrins and the yeast Wsc1 exhibit bidirectional signalling with the actin cytoskeleton. In mammals, actin cytoskeleton regulates integrin clustering, while actin dynamics is regulated by integrin-mediated adhesion [73]. Similarly, in yeast Wsc1 localization is mediated through actin cytoskeleton, while actin depolarization caused by cell wall damage is mediated through Wsc1 [74].

These cell wall mechanosensors in *S. cerevisiae* can be divided into two types – the WSC type that includes the genes *WSC1/SLG1* (*SLG1* is its standard gene name, but we will here use *WSC1* for consistency with the other WSC genes), *WSC2*, and *WSC3* and the MID type that includes the genes *MID2* and *MTL1*. The functional similarity between Wsc1 and integrins has been described in several studies [74–76]. The two mechanosensor types in yeast differ in the nature of their extracellular head groups, where the head group is a cysteine-rich domain in the WSC-type sensors but is an N-glycosylated asparagine in the MID-type sensors **(Fig. 3)**. The head group of the WSC-type sensors is presumed to be in contact with the glucan layer, while the head group of the MID-type sensors is presumed to be in contact with both the glucan and the mannoprotein layers in the cell wall [72]. The presumed physical interaction with the mannoprotein layer might give the MID-type mechanosensors the capacity to sense the thickness and elastic properties of the outer mannoprotein layer, as proposed by Heinisch and colleagues [72]. Because both sensor types can bind to the mannoprotein layer of the cell wall, they are believed to be redundant in sensing the thickness and elastic properties of the glucan layer.

Both types of yeast mechanosensors possess a cytoplasmic tail that communicates with downstream signalling components and thus allows cells to respond rapidly to external mechanical stress. The proposed model is that the mechanosensors' intracellular C-terminal tails interact with the GDP/GTP exchange factor Rom2 that subsequently activates the small GTPase Rho1 [77]. *RHO1* encodes an essential small GTPase in the Rho/Rac subfamily of Ras-like GTPases, and in *S. cerevisiae* Rho GTPases are also encoded by *RHO2, RHO3, RHO4, RHO5*, and *CDC42*, and of these at least Rho5 has also been shown to be activated by Wsc1 [78]. Rho1 regulates protein kinase C (encoded by *PKC1*), which is a protein serine/threonine kinase that is essential for cell wall remodelling during growth. Pkc1 is a homolog of the alpha, beta, and gamma isoforms of mammalian protein kinase C (PKC). Pkc1 then activates a basic three-protein kinase signalling module that integrates the MAPKKK Bck1, the redundant MAPKKs Mkk1 and Mkk2, and the MAPK Slt2.

The cellular roles of the mechanosensors in yeast have many similarities with integrins. Integrins in humans are involved in the establishment of cell polarity [79], the response to osmotic stress [80], actin cytoskeleton organisation [81], the response to heat stress [82], regulation of autophagy [83], aging [20], and the positive regulation of endocytosis [84]. In a similar manner, Wsc1 in *S. cerevisiae* is also involved in the establishment of cell polarity [74, 85], the response to osmotic stress [86], actin cytoskeleton organisation [74], the positive regulation of endocytosis [85, 87], the response to heat [74, 88], and the regulation of autophagy [89]. The mechanosensors also have an impact on chronological life span in yeast [90, 91]. In addition, both human integrins and yeast mechanosensors are members of the RHO-dependent signal transduction pathway that communicates downstream with the MAPK signalling pathway [37, 74, 77]. Wsc1 also has a dynamic localisation that resembles the polarised distribution of integrins in mammalian cells [76], where the polarised distribution for some of the mechanosensors is mediated through their endocytosis and recycling [92]. Moreover, human integrins protect cells from the genotoxic effects of bleomycin (a drug that degrades DNA) [93], and *wsc1* mutant yeast are hypersensitive to bleomycin [94]. Wsc1 acts upstream of Ste20 [95], which is required for pheromone-induced apoptosis [96], and upstream of Rho5, which plays a role in oxidative stress-induced apoptosis [97], and both of these observations suggest that Wsc1 might play a role in apoptosis in a similar manner as human integrins [98]. Taken together, *S. cerevisiae* possesses mechanosensors connected to the cell wall that in many ways are functional analogues to human integrins.

## THE YEAST CELL WALL IS A HIGHLY DYNAMIC STRUCTURE

The cell wall in yeast resembles the animal ECM, and similar to the ECM changes in the cell wall in yeast trigger different biological responses. The yeast cell wall determines cell shape, provides rigidity that counteracts the outward turgor pressure on the plasma membrane, protects the cells against external environmental injury, and acts as a diffusion barrier delimiting the periplasmic space. The yeast cell wall is a prominent structure that makes up 15–30% of the cellular dry weight [99, 100], has a thickness of 110–200 nm [101–103], and is mainly built from glucans (β-1,3-glucan and β-1,6-glucan), proteins (many of them being mannoproteins containing mannose), and chitin (N-acetylglucosamine polymers) [101]. An internal skeletal framework in the yeast cell wall forms a three-dimensional network of β-1,3-glucan molecules that surrounds the whole cell. This layer is primarily responsible for the mechanical strength of the cell wall. The β-1,3-glucan molecules are branched and they therefore have multiple nonreducing ends that can act as attachment sites for other components of the cell wall. Some of the mechanosensors impact on cell wall synthesis, i.e. deletion of *WSC1* results in a drastic decrease in the synthesis of β-1,3-glucan at the bud [104]. The skeletal glucan framework is strengthened close to the plasma membrane by chitin chains [105]. Chitin represents a minor component of the cell wall (only 1–3% of its biomass), but chitin is important for the proper function of the cell wall and is essential for fungal viability [106]. It has been shown that either overexpression of the mechanosensor Mid2 [88] or mutations that result in a defective cell wall [107, 108] cause hyper-accumulation of chitin. Chitin polymers are covalently cross-linked to the β-glucan network and are thought to contribute to the rigidity and physical strength of the cell wall [109]. On the outside of the β-1,3-glucan skeletal framework are mainly found β-1,6-glucan molecules that interconnect cell wall proteins with the central β-1,3-glucan framework [100].

**Figure 4:**
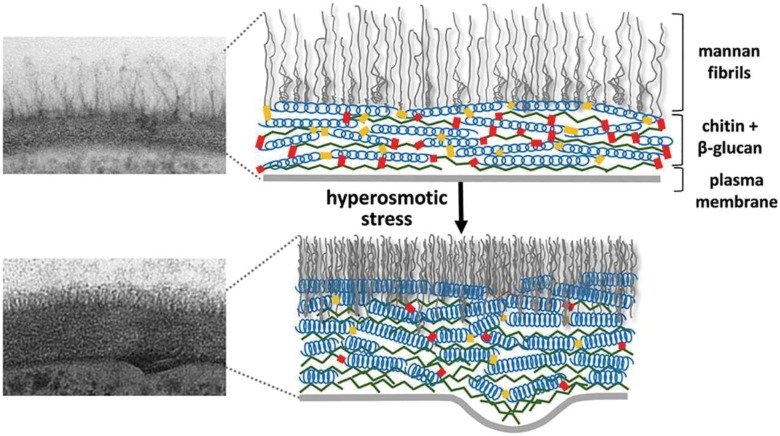
FIGURE 4: Cell wall structure before and after hyper-osmotic stress. The cell wall is shown as a transmission electron microscopy image (left) and as a schematic drawing (right). After osmotic dehydration (hyper-osmotic shock) the cell wall becomes much more compact and thicker. In the schematic drawing, this is indicated as the β-glucan helical structures acting like springs being compressed. This figure is from [150] and is published with permission from the American Society for Microbiology. Copyright © 2015, American Society for Microbiology.

The yeast cell wall is a highly dynamic structure, and its elasticity depends on a number of external and internal factors like nutrient source, temperature, and certain mutations; for example, cell wall elasticity can be increased by mutations that inactivate the CRH family of cell wall cross-linking chitin transglycosylases or can be decreased by overexpression of CRH family members [110]. It has also been observed that a sudden decrease in cell volume as a result of osmotic shock drives rapid increases in cell wall thickness. However, these changes in cell wall thickness are reversible, and quite rapidly the cell diameter and cell wall thickness return to normal when the osmotic stress is relieved. This behaviour seems analogous to springs being oriented parallel to the plasma membrane that are compressed and relaxed **(Fig. 4)**. Several molecular factors contribute to the elasticity of the *C. albicans* cell wall, and it was suggested that the helical conformation of β-glucans might permit their expansion and contraction [111]. In addition, slippage of locally aligned polymers over one another could promote the rapid changes that are observed in the dimensions of the glucan and chitin layers in the cell wall. An important factor for this elasticity is that the helical compression and polymer slippage are constrained by covalent crosslinks between cell wall polymers (chitin and glucans) as well as between these polymers and the cell wall proteins.

Inhibition of enzymes involved in the biosynthesis of cell wall components also leads to altered cell wall properties. For example, calcofluor white is a fluorescent agent that strongly binds to chitin [112] and interferes with chitin synthesis [112, 113]. The ATP analogue caffeine is also considered a drug that imposes cell wall stress [114]. Caffeine has been shown to inhibit the Tor1 kinase [115, 116] which results in activation of the CWI pathway leading to changes in the cell wall (see below). Caspofungin is an antifungal from the echinocandin family that inhibits β-1,3-glucan synthesis [117] and thus causes an increase in the β-1,6-glucan polysaccharide fraction and a partial reduction of β-1,3-glucan, both in *S. cerevisiae* and *C. albicans* [118]. All of these agents are frequently used in studies of the impact on cellular physiology and intracellular signalling from changes in the cell wall structure.

## THE YEAST CELL WALL IS CONNECTED TO THE ACTIN CYTOSKELETON

### Mechanosensors in *S. cerevisiae* and the link to actin

There is strong evidence that the cell wall and the actin cytoskeleton regulate/influence each other in yeast, and enzymatic removal of the cell wall (to form protoplasts) causes the actin cables to disappear and the actin patches to become evenly distributed over the cell surface [119]. The cell wall of *S. cerevisiae* displays nano-mechanical oscillating movements that are suggested to be caused by molecular motors in the cell [120], and such movement means that the cytoskeleton needs to be connected to the cell wall to allow force transfer to the cell wall [121]. Control of the actin cytoskeleton is also an important element in the compensatory response to weakening of the cell wall by altered external conditions, for example, by heat shock [71].

Like mammalian integrins, the yeast mechanosensor Wsc1 both controls and responds to the actin cytoskeleton [74]. Wsc1 acts as a regulator of rearrangements of the actin network during conditions of cell wall expansion and membrane stretching, and it is also a component of the cap, a complex structure located at the bud tip that is essential for the regulation of polarity of the actin cytoskeleton [122]. The actin patches are redistributed from the polarised growth sites to the cell periphery during cell wall stress, and this depolarisation is mediated by Wsc1 [74]. The actin network can also undergo depolarisation following hypo-osmotic shock, but at longer times of hypo-osmotic conditions repolarisation of actin occurs. Interestingly, *wsc1* mutants show a marked delay in the repolarisation of the actin cytoskeleton after an osmotic shift, and about 40% of *wsc1* mutant cells are still depolarised at 2 h after a hypo-osmotic shift [71]. The fact that loss of Wsc1 affects actin repolarisation suggests that Wsc1 might be an essential anchor site for actin. However, the Wsc1 protein does not appear to bind directly to actin, so how then is Wsc1 linked to the actin cytoskeleton?

### Talin homologues in yeast connect Wsc1 to actin

**Figure 5:**
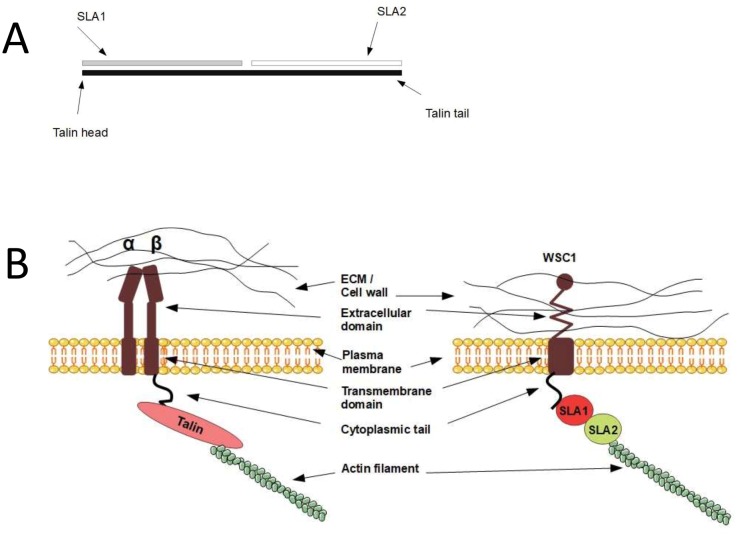
FIGURE 5: Sequence and functional analogy between human talin and yeast Sla1 and Sla2. **(A)** Yeast Sla1 and Sla2 are homologous to the N-terminal and C-terminal parts of human talin, respectively. **(B)** A hypothetical model where Sla1 and Sla2 bind and act in analogy to human talin in connecting the mechanosensors to the actin cytoskeleton. Left, human and integrin-talin; right, yeast and mechanosensor-Sla1/Sla2.

In mammals, integrin binds to the actin cytoskeleton via adapter proteins, of which talin is the most prominent **(Fig. 1)**. The talin head binds to the cytoplasmic domain of integrin [123], while its tail binds the actin cytoskeleton [124]. In *S. cerevisiae*, the adapter protein Sla2 has been reported to possess a domain with talin homology [125]. Similarly to mammalian talin, Sla2 binds to actin via an I/LWEQ domain, and the deletion of this domain in Sla2 leads to defects in actin cytoskeleton morphology [126]. In contrast, overexpression of Sla2 is lethal and leads to the formation of thick actin cables [127]. Different studies suggest that Sla2 functions between the actin cytoskeleton and the endocytosis machinery [127–130]. In addition, Sla2 was identified together with Sla1 as synthetic-lethal with the actin-binding protein Abp1 [125], indicating related functions of Sla1 and Sla2. The protein Sla1 is important for proper localisation of Sla2 [131]*,* and Sla1 can physically interact via its SHD1 domain with Wsc1 [76]. Sla1 binds Wsc1 to direct it into endocytosis [76], and this function of Sla1 is reminiscent of talin, which binds to integrin to keep it in an active conformation during endocytosis [132]. The fact that Sla1 and Sla2 physically interact [133, 134] and function together suggests that they might act as a heterodimer that functions as an adapter between the actin cytoskeleton and Wsc1 in an analogous manner to mammalian talin. Surprisingly, by aligning the protein sequences of Sla1, Sla2, and human talin, we found that Sla1 aligns to the N-terminal part of human talin, while Sla2 aligns to the C-terminal part **(Fig. 5A)**. Even though the sequence similarity is rather weak, this observation suggests that human talin is a fusion gene, i.e. a “Rosetta stone”, of Sla1 and Sla2. Taken together, yeast Sla1 and Sla2 might function together in a similar manner to human talin and physically connect Wsc1 to the actin cytoskeleton **(Fig. 5B)**.

## MECHANOSENSORS AND INTRACELLULAR SIGNALLING

Many components of integrin-dependent signalling in humans are conserved in yeast. For example, Sla1 and Sla2 are homologues of human talin (see above), the yeast Pxl1 is a homologue of human paxilin [135], Pfy1 is a homologue of human profilin [136], Tep1 is a homologue of PTEN [137], and Las17 is a homologue of WASP [138]. In mammals, the LIM domain of paxilin targets the protein to FACs [139], while in yeast it has been shown that the LIM domain in the paxilin-homolog Pxl1 targets the protein to sites of polarised growth [135]. Las17, Sla1, and Sla2 are components of the actin cortical patch in yeast [131, 140], which suggests that the actin cortical patch might act as a yeast functional analogue to the human FAC.

Mammalian down-stream signalling pathways that receive input from integrins contain signalling components that have homologues in yeast. For example, the human ERK2 MAPK is a homolog of Kss1 in yeast, p38 MAPK is a homolog of Hog1, and ERK5 is a homolog of Slt2. The CWI pathway, which is the main signalling pathway responsible for maintaining cell wall homeostasis, has been extensively studied in *S. cerevisiae* [114]. As indicated above, the mechanosensors act as upstream sensors for the CWI pathway. A key component in the CWI pathway is the MAPK Slt2, which activates transcriptional responses to counteract cell wall stress mainly through phosphorylation of the Rlm1 transcription factor. Activated Slt2 also has a non-catalytic domain with which it forms a complex with Swi4/Swi6 and activates the *FKS2* promoter (Fks2 is the catalytic subunit of β-1,3-glucan synthase) [141].

## THE YEAST CELL WALL AND MECHANOSENSORS AS MEDIATORS OF VARIOUS EXTERNAL CONDITIONS

How yeast cells sense and respond to external perturbations are vital fields of investigation. Here we will give some examples in which the cell wall and the integrin functional analogues, the mechanosensors, play important roles in initiating intracellular signalling in response to changes in the external conditions, and we will provide connections to integrin-linked responses in mammals.

### The heat shock response

*S. cerevisiae* responds to changes in temperature through drastic modifications of gene expression, and roughly 10% of all genes display significant changes in expression in response to elevated temperature [142]. Central to this transcriptional response in yeast is the heat shock transcription factor Hsf1. In addition to the effects of heat shock on internal cellular processes, thermal stress also appears to impact the cell surface. It has been shown that heat shock induces structural weakness in the cell wall that leads to activation of signalling via a putative cell wall elasticity/stiffness sensor [75, 143]. In line with this, studies have reported on the importance of the yeast mechanosensors in the response to heat [74, 88]. It was found that Wsc1, Wsc2, and Mid2 – and the GDP/GTP exchange factor Rom2 that interacts with these cell wall mechanosensors – are all multicopy suppressors of a temperature-sensitive *hsf1* mutation [144]. Activation of the Pkc1 protein was furthermore shown to be necessary for suppression of the *hsf1* mutation. This indicates that Hsf1 may act in parallel with the mechanosensors and Pkc1 to regulate cell wall remodelling in response to heat shock. In addition, overexpression of several other enzymes involved in cell wall organisation, like exo-1,3-β-glucanase (Exg1) and β-1,6-glucan synthase (Kre6), were also shown to suppress the temperature sensitivity of the *hsf1* mutant. In addition, the *hsf1* mutant was also suppressed in sorbitol medium that provides osmotic support, further highlighting the presence of a cell wall defect in the *hsf1* mutant. It has also been reported that the mechanosensor triple mutant *wsc1Δwsc2Δwsc3Δ* is more sensitive to heat shock compared to wild type [145]. Taken together, there is ample genetic evidence that heat shock affects the organisation of the cell wall and that a proper cellular response to heat is dependent on the mechanosensors and down-stream signalling via Pkc1.

Heat shock has been shown to modulate several important properties of the yeast cell wall [146–148]. A shift from 30°C to 42°C induces the formation of a concentric circular ring on the yeast cell wall that appears after 20 minutes and reaches a diameter of 2–3 μm roughly 1 h after the temperature shift [146]. It has been shown that active cell growth is essential for the formation of the circular structure, and its appearance is accompanied by an increase of chitin in the cell wall and an increase in cell wall stiffness [146]. Heat shock also causes an increase in the β-1,6-glucan polysaccharide fraction and a partial reduction of β-1,3-glucan, both in *S. cerevisiae* and *C. albicans* [118]. All of these heat-induced cell wall changes might be detected by the mechanosensors and transmitted to intracellular signalling pathways, ultimately leading to drastic changes in gene expression and cell physiology/heat tolerance. We thus propose that the heat shock response in yeast is partly sensed by the mechanosensors, detecting heat-induced changes in the cell wall properties. Two possibilities arise: the first one is that heat affects membrane fluidity and that this change can be detected by the mechanosensors; membrane fluidity changes lead to a change in cell wall elasticity [149]. The second possibility is that heat affects the activity of pre-existing wall-editing enzymes, which leads to a change in cell wall elasticity. An analogy to this hypothesis is that osmostress causes an immediate change in cell wall elasticity by affecting wall editing enzymes [150]. The model is that upon heat shock and subsequent changes to the cell wall, Wsc1, Wsc2, and Mid2 activate Rom2 to promote GTP loading of Rho1, which in turn activates Pkc1 in the CWI pathway and leads to gene expression changes and cell wall remodelling.

### The osmotic response

There is a large body of literature regarding osmostress on yeast, with extensive studies on several of the signalling pathways involved [151] and the multitude of genes and proteins that take part in the resulting physiological changes [152–155]. However, less attention has been devoted to the immediate morphological effects of the yeast cell wall in response to hyper-osmotic shock [156].

The yeast cell wall structure is modulated by osmostress. In *C. albicans*, cell wall remodelling enzymes change the cell wall elasticity, and a more elastic cell wall can be generated within seconds after osmotic stress [111]. Cells shifted to a hyper-osmotic culture medium exhibit fluorescent patches distributed irregularly over the cell surface when stained with cell-wall specific markers (e.g. calcofluor). These patches are proposed to be the result of redistribution of the pre-existing cell wall material into plasma membrane invaginations as a result of plasmolysis caused by osmotic dehydration [156]. Interestingly, this immediate osmo-response is not dependent on the HOG signalling pathway because *hog1* and *pbs2* mutant cells respond with similar cell wall patchiness as wild type cells. Yeasts growing on different carbon sources are differently sensitive to hyper-osmotic shock [157]. For example, lactate-grown yeast cells are more resistant than glucose-grown cells to osmotically induced volume changes, potentially due to structural differences in their respective cell walls [150]. The elevated resistance of lactate-grown cells correlates with reduced cell wall elasticity, as reflected in slower changes in cell volume following hyper-osmotic shock, and this is believed to lead to enhanced survival. These osmostress-induced changes in cell wall elasticity have also been confirmed by atomic force microscopy measurements of wild type and mutant cells and have been shown to reflect changes in the Young's modulus [150].

The MAPK Hog1 is the final component in the main signalling pathway that responds to hyper-osmotic stress in yeast. The human Hog1 orthologue p38 MAPK can functionally replace Hog1 in yeast [151], and the activation of p38 MAPK in response to mechanical stress is mediated through integrin [158]. In addition, cell swelling caused by starvation in human cells is sensed by integrin, which activates p38 MAPK [83, 159]. So, could it be that the upstream activator of Hog1 in *S. cerevisiae* might be an integrin functional analogue? Indeed, the HOG pathway in yeast is activated by a large number of external stressors, including osmostress [160], low pH [161], zymolyase digestion of the cell wall [162], and microgravity [163], all of which affect the mechanical properties of the cell wall or the membrane-cell wall interaction. The HOG pathway in yeast is activated by two branches; the first is via Sln1 and the second is via Sho1, Hkr1, and Msb2 [164]. In the latter, the membrane protein Sho1 functions as an adapter in recruiting the kinases responsible for signalling in response to osmostress [165]. It has been shown in high-throughput studies that resistance to hyper-osmotic stress is decreased in *WSC1* deletion mutants [166] and *WSC2* deletion mutants [167]. The CWI pathway is activated and plays its main role in response to hypo-osmotic shock [168]. However, there are also reports of cooperative crosstalk between the HOG and CWI pathways suggesting that they jointly regulate the cell's response to zymolyase-induced cell wall damage. The HOG pathway shares some functions with the CWI pathway, and both are involved in cell wall biogenesis and are activated by hyper-osmotic shock, heat shock, oxidative stress, and zymolyase stress [169].

In conclusion, the cell wall and the mechanosensors appear to play important roles in several aspects of the osmo-response and to exert their effects through the HOG and CWI signalling pathways.

### The starvation response

There are different signalling pathways in yeast that sense nutrient levels. There are several plasma membrane components involved in these processes, including receptors (Gpr1 and its associated down-stream Gα protein Gpa2), transporter-like sensors (Snf3 and Rgt2), and combined transporters and receptors (transceptors, e.g. Gap1). These nutrient sensors transmit their signals through various signal transduction pathways that involve central protein kinases like PKA, TORC1, and Sch9 [170]. In addition, both cyclic AMP (cAMP) and protons act as second messengers, and there are internal proteins, like the hexokinase Hxk2 and the AMP-activated S/T protein kinase Snf1, for intracellular sensing and signalling of the availability of glucose. The deletion of SNF1 causes sensitivity to cell wall stress agents and causes reduction in cell wall thickness in *S. cervisiae* [171]. Thus, the network of molecular players in the response to nutrients is rather complex. We will not cover all of the details of these pathways, but refer the reader to several recent extensive reviews that provide excellent overviews of this interesting field [170, 172, 173]. Here we will focus on additional aspects of nutrient signalling where the cell wall and the mechanosensors appear to be involved.

In mammals, integrins mediate cAMP signalling through a G-protein dependent pathway [174] and activate the cAMP pathway through the Gαs protein at FACs [175]. Gpa2 is the Gαs homologue in *S. cerevisiae*, and overexpression of Gpa2 in yeast increases cAMP levels [176]. Thus, Gpa2 in yeast can activate the cAMP pathway similarly to its mammalian homologue. An integrin-like mechanistic connection between the mechanosensors and cAMP production in yeast seems plausible, and it has been reported that Gpa2 physically interacts with the mechanosensors Wsc2, Wsc3, and Mid2 [133]. The central component in the glucose-sensing pathway in yeast is the RAS/PKA system [177]. cAMP is synthesised through adenylyl cyclase, the activity of which is stimulated by the GTP-bound Ras1 and Ras2 proteins. However, despite extensive studies regarding the above-mentioned receptors and signalling components, the mechanism by which glucose activates the RAS proteins is still not fully understood [172, 173]. A potentially critical protein in glucose signalling is Gpa2, which along with its upstream receptor Gpr1 participates in the activation of PKA through the stimulation of adenylyl cyclase activity and the subsequent increase in cAMP production. However, the importance of Gpr1 and Gpa2 in glucose sensing has also been strongly questioned [173]. Several lines of evidence indicate that Gpr1 does not serve as a primary mediator in the acute response of cells to glucose; for example, deletion of Gpr1 or Gpa2 has no effect on the transcriptional response to glucose addition [177]. To further complicate things, it is clear that different mechanisms are involved in short-term and long-term responses to nutrient limitations, but the specifics of these mechanisms are not fully understood [172].

We propose here that the cell wall and the yeast mechanosensors might be part of yet another sensory/signalling pathway, compared to the ones described above, that is activated during nutrient starvation. The Mtl1 mechanosensor appears to be important in this respect, and it has been shown that Mtl1 activates the CWI pathway in response to glucose starvation [178]. Recent data indicate that Rho5, which is down-stream of the mechanosensors, might be involved in the response to glucose starvation [179], which gives further support for the involvement of the mechanosensors in nutrient sensing. Further mechanistic insights into this nutrient-sensing hypothesis comes from the fact that Wsc1, Wsc2, Wsc3, and Mtl1 have all been identified as inhibitors of the RAS2-cAMP pathway [145, 180]. Petkova *et al.* showed that the *mtl1* deletion mutant is deficient in the transcriptional induction of specific stress genes upon glucose deprivation [178]. These authors also observed that higher levels of the stress-induced transcription factor Msn2 are sufficient to rescue this glucose-dependent induction defect in the *mtl1* deletion mutant in response to glucose starvation. Because the nuclear localisation of Msn2 is controlled by the RAS/PKA pathway and because *mtl1* mutant cells show higher levels of cAMP compared to wild type, it was suggested that Mtl1 impacts on Msn2 activity by controlling cAMP levels through the down-regulation of Ras2. In line with this, Verna *et al.* found that deletion of *RAS2* could rescue the heat shock-sensitive phenotype of the *wsc1*Δ strain, i.e. *RAS2* and *WSC1* showed a genetic interaction indicating that they are functionally linked [145]. Based on various experiments, including overexpression of Wsc1 in different mutant strains, the authors concluded that Wsc1 acts downstream of Ras1/2, perhaps modulating the activity of adenylate cyclase or the activity of PKA [145]. Thus, even if there are somewhat different working hypotheses for the mechanistic connection between the mechanosensors and cAMP/PKA, i.e. directly via inhibition of RAS proteins or of Gpa2, it is clear that the mechanosensors affect cAMP levels and PKA activity. Future experiments are needed to conclude which of the two mechanisms is the most prominent or if both connections exist and are equally important.

Another interesting observation is that the antifungal drug caspofungin, which inhibits the β-1,3-glucan synthase and in that way affects the cell wall, activates the CWI pathway involving Wsc1, Slt2, and Rom2 and inhibits the RAS/PKA pathway, the latter by decreasing cAMP levels (the decrease in cAMP levels was substantial and close to the cAMP decrease observed in a *ras2*Δ mutant [108]). The effects on RAS/PKA from caspofungin treatment were independent of the MAPK Slt2, but were dependent on Wsc1. This indicates that Wsc1 may transduce the change in the cell wall made by caspofungin through RAS/PKA, and that the effect on RAS/PKA is not an indirect consequence of MAPK signalling. Garcia *et al.* found that caspofungin treatment reduced levels of Ras2-GTP (the active form of Ras2) to similar levels as seen under conditions of glucose starvation [108]. Taken together, all of these results strongly argue in favour of a mechanistic connection between the mechanosensors and the RAS proteins leading to the de-activation of the RAS/PKA pathway in yeast during nutrient-poor conditions.

Further support for the involvement of the yeast cell wall in nutrient sensing comes from the fact that the chemical composition of the cell wall is modulated under various nutritional regimes, leading to changes in cell wall properties. When grown under NH_4_ limitation, the *S. cerevisiae* cell wall contains only half as much protein as cell walls from cells grown under glucose-limiting conditions [181]. In addition, under nitrogen limitation the cell walls are rigid and their inner layers look spongy under an electron microscope, while under glucose limitation cell walls are more elastic and appear more compact. The yeast *Kluyveromyces lactis* has a cell wall thickness of 64 nm when the carbon source is glucose, but it is 105 nm thick when the carbon source is ethanol. In addition, the cell walls of *K. lactis* grown on ethanol have a chemical structure that is more sensitive to breakdown by zymolyase [182]. Also, in *C. albicans* there are reports of changes in cell wall thickness and elasticity in response to changes in carbon source, and the thickness of the inner cell wall layer decreased from 75 nm to 22 nm when the carbon source was changed from glucose to lactate [111]. It has also been shown that cells grown on lactate display higher elasticity than cells grown on glucose [150]. We propose that these nutrient-induced changes in cell wall properties like thickness and elasticity are sensed by the mechanosensors that together with the cell wall form a sensory system for nutrient availability. In our model, the mechanosensors sense these nutrient-imposed cell wall changes and transmit signals intracellularly to activate various signalling pathways, in particular the RAS/PKA pathway.

### The filamentous growth pathway

External factors can also induce developmental programs in yeast. In particular, starvation for some nutrients stimulates different developmental responses – nitrogen starvation induces mating [183], carbon starvation induces haploid and diploid yeast to enter the quiescent state [184], carbon starvation can stimulate invasive growth in haploid yeast [185], in diploid cells nitrogen starvation induces pseudohyphal growth, and starvation for both nitrogen and carbon stimulates sporulation [186, 187]. However, it is still not completely clear how nutrient limitations cause all these developmental responses.

Filamentous growth is a regulated developmental response characterised by cell elongation, unipolar budding, physical attachment of mother and daughter cells, and increased adhesion to and invasion of growth substrates. Filamentous growth is a complex process which is regulated by many different signalling pathways and transcription factors [188]. The current model of filamentous growth signalling suggests the two mucins Msb2 and Hkr1 as the upstream components in the filamentous growth MAPK (FG-MAPK) pathway, that transmit the signal via Sho1 and through a signalling cascade involving Cdc24, Cdc42, Ste20, Ste50, Ste11 and Ste7 to reach the MAPK Kss1, which activity leads to the transcription of filamentous growth genes. In *S. cerevisiae*, filamentous differentiation is also positively correlated with the activity of the cAMP-PKA pathway, and increased intracellular cAMP levels or PKA activity result in increased filamentous growth [188]. Both the cAMP-PKA pathway and the FG-MAPK pathway are capable of inducing pseudohyphal growth in *S. cerevisiae*, and many important transcription factors and other targets are regulated in parallel by both pathways. Both cAMP-PKA signalling and the FG-MAPK pathway are regulated by Ras2, but it is not fully clear how Ras2 is activated by upstream components that respond to nutrient availability. Filamentous growth is also regulated by Tor1, and the Tor1 inhibitor rapamycin inhibits filamentous growth. This response is mediated through TAP42, a phosphatase in the TOR pathway, and the overexpression of Tap42 in cells treated with rapamycin restored filamentous growth [189]. In addition to the factors mentioned above, both the AMP-activated S/T protein kinase Snf1 and the transcription factor Rim101 take part in regulating filamentous growth [188].

A connection between filamentous growth and the mechanosensors has been established [190], and overexpression of mechanosensor genes was shown to induce expression of a filamentous growth-reporter (the transcription factor Fus1). In addition, overexpression of *MID2* and *WSC2* increased invasive growth, and deletion mutants of the mechanosensors (*wsc1*Δ*, wsc2*Δ, and to a lesser degree *mid2*Δ) exhibited defects in invasive growth. Because nutrient limitation alters the cell wall thickness and elasticity (see above), these changes in the cell wall might be part of the stimulus for the induction of filamentous growth. Haploid and diploid cells differ in terms of cell volume [191] and the thickness of their cell walls (4.18 µm and 5.3 μm, respectively) [191, 192]. The difference in cell wall thickness and cell volume might have an impact on the distribution and the binding affinity between the wall mechanosensors and the cell wall and thus trigger the developmental changes. This hypothesis thus suggests that filamentous growth might also be affected by any factor that perturbs the cell wall dynamics independent of nutrient starvation. Indeed, in *S. cerevisiae* grown in rich medium with an adequate supply of nitrogen and other nutrients, both pseudohyphal formation and invasive growth (both of which are aspects of filamentous growth) were stimulated under certain stress conditions that affect the cell wall structure, such as increased temperature (37°C), osmotic stress (1 M NaCl), and drugs that perturb the glucan layer of the cell wall (i.e. Congo red) [187]. Conversely, it has also been shown that invasive growth is reduced in starved yeast cells under microgravity conditions, where microgravity decreases the mechanical stress on the cell wall [193].

In mammals, integrin regulates the activation of the ERK pathway in response to mechanical stress [194], and it has been shown that integrin-ECM interactions regulate ERK1/2 activity [195]. In *S. cerevisiae*, pseudohyphal growth is mediated by the KSS1 pathway [196], where the MAPK Kss1 has strong sequence similarity with human ERK1. A constitutively active form of human ERK1 induced pseudohyphal growth in *S. cerevisiae* [197].

**Figure 6:**
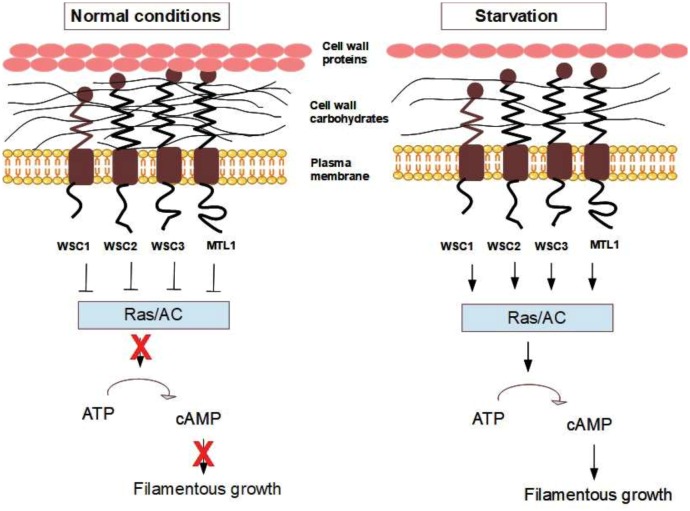
FIGURE 6: Hypothetical model for how changes in the cell wall are sensed by mechanosensors and transmitted to RAS and lead to filamentous growth. Starvation induces changes in the cell wall organisation, and this leads to changes in wall elasticity that are sensed by the mechanosensors. The mechanosensors act as inhibitors of Ras1/2 and/or adenylate cyclase (AC) under normal growth conditions. Starvation relieves this inhibition resulting in increased levels of cAMP and increased PKA activity, which triggers filamentous growth.

The current model states that the above-mentioned sensors and signalling mechanisms, e.g. Msb2, FG-MAPK, RAS/PKA and TOR, respond to nutrient limitations and trigger filamentous growth and in parallel change the cell wall [188]. However, we here propose an alternative model for nutrient-limitation signalling via cell wall changes and the mechanosensors during the initiation of filamentous growth. Yeast cells undergo filamentous growth when cAMP levels and PKA activity are high [198]. As indicated above, cAMP is produced by the enzyme adenylate cyclase, which is activated either by Gpa2 or Ras2. It has been shown that Gpa2 controls cell differentiation into filamentous growth in response to glucose starvation [199], and it has been reported that Gpa2 physically interacts with the mechanosensors Wsc2, Wsc3, and Mid2 [133]. Both the Wsc2 and Mid2 mechanosensors were identified as regulators of filamentous growth [190]. Moreover, overexpression of either Wsc1 and Wsc3 increased filamentous growth [200]. Together, these studies suggest that pseudohyphal growth in *S. cerevisiae* is probably at least partly regulated by an integrin-analogous signal in response to structural changes in the cell wall. In our hypothetical model **(Fig. 6)**, the mechanosensors Wsc1, Wsc2, Wsc3, and Mtl1 (the ones identified as inhibitors of the RAS2-cAMP pathway [145, 180]) inhibit RAS/adenylate cyclase under normal conditions, while changes in cell wall elasticity caused by starvation or mechanical stress might change the conformation of the cell wall sensors and remove this inhibition. In our model, this leads to the activation of the adenylate cyclase enzyme, increased cAMP levels, enhanced PKA activity, and the activation of the filamentous growth cascade.

## CONCLUSIONS AND FUTURE CHALLENGES

In this review we discuss a general principle that seems adopted by all forms of life – stretching of either the cell wall or the plasma membrane generates a mechanical force on the extracellular part of a mechanosensor, and this stimulus is transmitted into a conformational change within the intracellular, cytoplasmic tail of the sensor and a subsequent activation of downstream signalling pathways and re-orientation of the actin cytoskeleton [201]. This is the main functional theme of the mammalian integrins. It is clear from sequence similarity that integrins exist throughout the metazoans, as well as in some unicellular organisms. Integrin-like proteins have been detected in fungi, but it should be stressed that these integrin-like proteins in fungi have no clear overall sequence similarity to their mammalian counterparts but possess shorter integrin-like motifs. They do have an integrin-like design, and thus they appear not to be evolutionarily related to integrins but to be functional analogues and to contain parts that have integrin-like features.

In the model yeast species *S. cerevisiae* there is no protein with sequence similarity to mammalian integrins. However, we propose here, as some other authors before us have done, that the mechanosensors in yeast are integrins in disguise – they are integrin functional analogues. We exemplify this with a number of experimental studies where the WSC-type and MID-type mechanosensors in yeast have been shown to resemble the integrins, both in terms of their mechanistic features and in their overall phenotypic consequences. We also propose that the cell wall is a prominent cellular feature of yeast that is involved in sensing/signalling pathways for a number of external factors. We believe that this is a generally neglected aspect of yeast physiology, and we propose that more in-depth studies are needed of the impact of the cell wall in relation to all aspects of yeast biology. In a hypothetical model, we propose that nutrient limitations modulate cell wall elasticity, which is sensed by the mechanosensors and results in developmental changes such as the induction of filamentous growth.

We also propose that the general importance of the mechanosensors in a number of central responses in yeast might have been missed because of functional redundancy among these proteins. Overall, there are five different genes encoding these mechanosensors in *S. cerevisiae* and few, if any, studies have really explored their functions in isolation. In this respect, it would be valuable to make penta-deletion mutants studying one mechanosensor at the time by reintroducing them individually. In the context of redundancy, it is interesting to note that other yeast species also harbour multiple redundant mechanosensor that complicates their functional characterisation, e.g. the milk yeast *K. lactis* has three mechanosensor paralogues [202]. In addition, the functions of the paralogues will most likely also be partly unique, where the different lengths of their extracellular domains might provide specificity in sensing different types of changes in cell wall elasticity and thickness. Finally, we would like to emphasise that the models proposed here, e.g. sensing glucose limitations, do not exclude the importance of other already well-known signalling pathways, but rather that they act in parallel and/or cooperate with each other.

We thus propose that there is still a lack of understanding of the cellular roles and molecular functions of the yeast mechanosensors. There is a long list of studies that can be proposed, and we will here only indicate some important ones. The Sla1 and Sla2 connection to human talin is interesting. Why is the long human talin protein split into two genes in yeast? Can the human talin protein compensate for the double mutant *sla1*Δ*sla2*Δ in yeast? What would happen if one makes a fusion protein of *SLA1* and *SLA2* to form a protein more in line with human talin? Will that fusion hamper some of the functionality that these two proteins individually take part in in yeast? In line with this, we also believe it would be interesting to examine the dynamics of the actin cytoskeleton in response to mutations in *SLA1* and *SLA2*. In particular, it would be revealing to study if the oscillatory movements observed by Pelling *et al.* [120] persist in *SLA1* and *SLA2* mutations or in WSC/MID gene deletions. These studies could either be conducted on the individual single deletion mutants of the mechanosensors or in the penta-deletion background expressing one of them at the time. These oscillatory studies would be interesting in order to get more mechanistic and functional information regarding the mechanosensor-actin connections and because the oscillatory phenomenon is still not fully understood.

Finally, another interesting research avenue would be to study the mechanosensors in relation to the cell cycle, and the presence of a “cell wall integrity checkpoint” to control cell cycle progression in relation to cell wall perturbations has been described (reviewed in [203]). A main feature of pseudohyphal growth is the delay in the G2/M phase, which allows daughter cells to grow to the same size as the mother cell before septum formation and cell separation [204]. We propose that this response might be regulated by the cell wall mechanosensors. Interestingly, defects in cell wall synthesis cause G2/M arrest, and this arrest in the cell cycle requires Wsc1 [205]. In mammals, integrin-mediated cell adhesion to the ECM regulates G2/M arrest [206]. We foresee that studies along these lines will deepen our mechanistic understanding of the connections between the cell cycle and the mechanosensors in yeast and will also have relevance for the understanding of the connection between integrins and the cell cycle in humans.

## Abbreviations:

cAMP: – cyclic AMP,
CWI: – cell wall integrity,
ECM: – extracellular matrix,
FAC: – focal adhesion complex,
FAK: – focal adhesion kinase,
MIDAS: – metal ion-dependent adhesion site,
VWA: – von Willebrand A.
